# Ischaemic stroke induced by neurocysticerosis, presenting as a clinical and radiological dilemma

**DOI:** 10.1259/bjrcr.20150254

**Published:** 2016-07-28

**Authors:** Rabia Hasan, Alberto Iaia, Carlos Flores

**Affiliations:** Department of Radiology, Christiana Care Health System, Newark, DE, USA

## Abstract

Neurocysticercosis (NCC) is a central nervous system parasitic infection with various clinical presentations, rarely manifesting as an acute stroke. The radiological appearance of this disease entity may be non-specific, at times mimicking an intracranial neoplasm. Early diagnosis requires a high index of suspicion. Serological testing is helpful and, if utilized early, can reduce the morbidity associated with invasive diagnostic techniques. We describe a case of a 32-year-old previously healthy male who presented with neurological deficits. A cystic lesion in the right sylvian cistern was noted, initially identified as a benign arachnoid cyst. The patient's symptoms rapidly progressed to an acute stroke. Follow-up imaging including an MRI of the brain demonstrated a right middle cerebral artery territory infarct adjacent to the cystic lesion, which had been diagnosed as an arachnoid cyst on an initial CT scan. Appearance of the cystic lesion on MRI, however, was concerning for a brain neoplasm or an abscess. Given the contiguity of the cystic mass to the right middle cerebral artery, it was suggested that the mass was the likely aetiology of the patient's symptoms. A stereotactic biopsy of the cystic lesion was performed and revealed it to be NCC. The hospital course was complicated by intracranial hypertension and cerebral oedema requiring craniectomy. Our case highlights the importance of considering NCC in the differential diagnosis of stroke in patients coming from endemic regions, especially in younger patients lacking the usual risk factors for cerebrovascular disease.

## Summary

Neurocysticercosis (NCC) is a central nervous system parasitic infection with various clinical presentations, rarely manifesting as an acute stroke. The radiological appearance of this disease entity may be non-specific, at times mimicking an intracranial neoplasm. Early diagnosis requires a high index of suspicion. Serological testing is helpful and, if utilized early, can reduce the morbidity associated with invasive diagnostic techniques. We describe the case of a 32-year-old previously healthy male who presented with neurological deficits. A cystic lesion in the right sylvian cistern was noted, initially identified as a benign arachnoid cyst. The patient’s symptoms rapidly progressed to an acute stroke. Follow-up MRI of the brain demonstrated a right middle cerebral artery (MCA) territory infarct adjacent to the cystic lesion, which had been diagnosed as an arachnoid cyst on an initial CT scan. Appearance of the cystic lesion on MRI, however, was concerning for a brain neoplasm or an abscess. Given the contiguity of the cystic mass to the right MCA, it was suggested that the mass was the likely aetiology of the patient’s symptoms. A stereotactic biopsy of the cystic lesion was performed and revealed it to be NCC. The hospital course was complicated by intracranial hypertension and cerebral oedema requiring craniectomy. Our case highlights the importance of considering NCC in the differential diagnosis of stroke in patients coming from endemic regions, especially in younger patients lacking the usual risk factors for cerebrovascular disease.

## Case presentation

**Figure 1.**
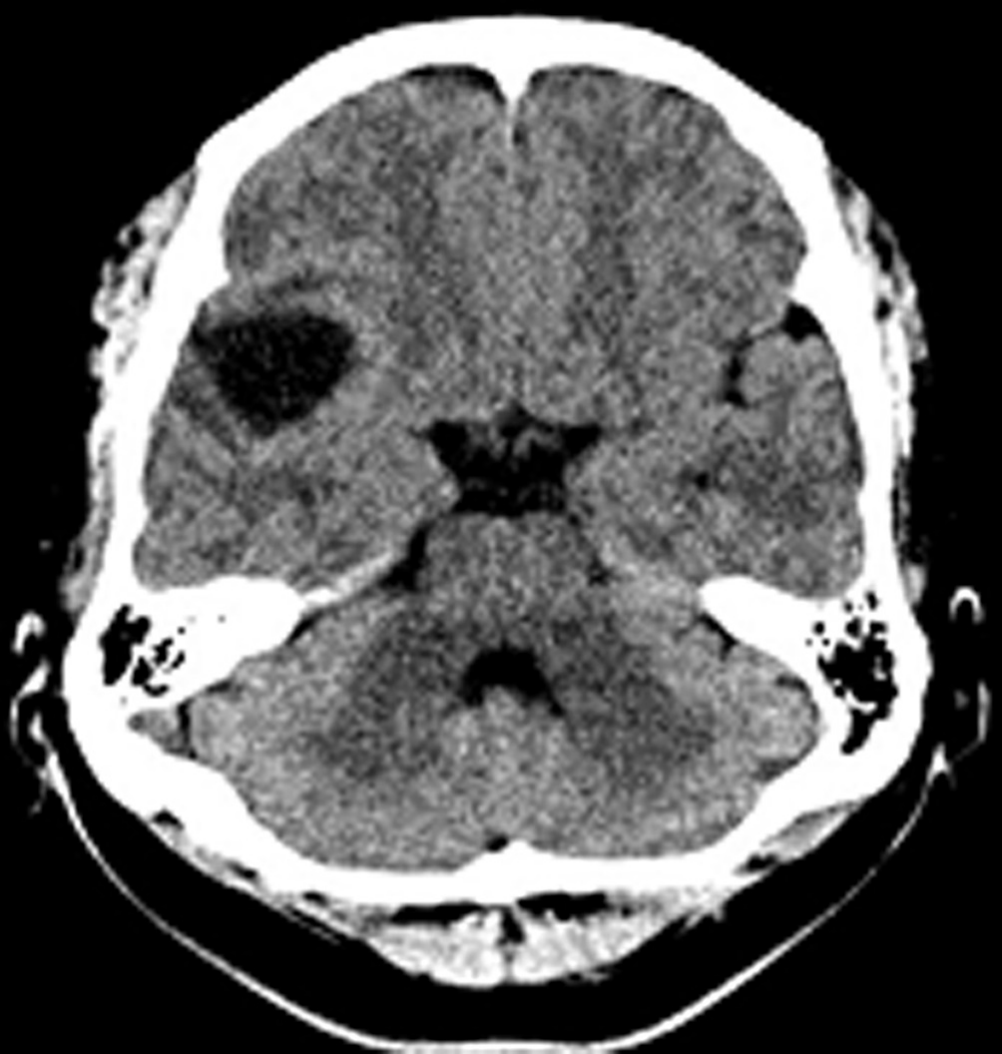
Non-enhanced CT scan of the head demonstrates a hypodense cystic lesion in the right sylvian cistern.

A 32-year-old Mexican immigrant male with no significant past medical history presented to the emergency department with an acute onset of facial tingling and headaches. On physical examination, he was afebrile, normotensive and had no signs to suggest any focal neurological deficits. Basic metabolic panel and complete blood count examinations were within normal limits. Head CT imaging demonstrated a cystic lesion in the right sylvian cistern, suspected to represent an arachnoid cyst (Figure 1). The patient was discharged after discussion with neurology, with a presumptive diagnosis of migraine with aura.

Subsequently, within 12 hours of discharge, the patient returned with left upper extremity weakness, left facial numbness and speech impairment. Physical examination revealed a left facial droop, left upper extremity weakness and dysarthria. A detailed review of symptoms was otherwise negative. No personal or family history of cerebrovascular events or risk factors was present.

## Imaging findings

**Figure 2.**
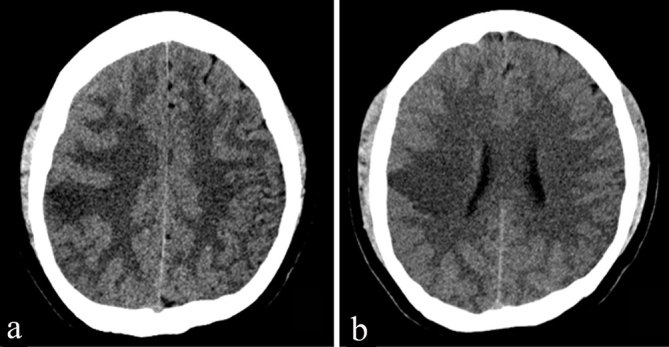
Non-enhanced CT scan of the head (a, b) demonstrating parenchymal hypodensity extending to the cortical surface along the right middle cerebral artery distribution, consistent with an acute infarct.

**Figure 3.**
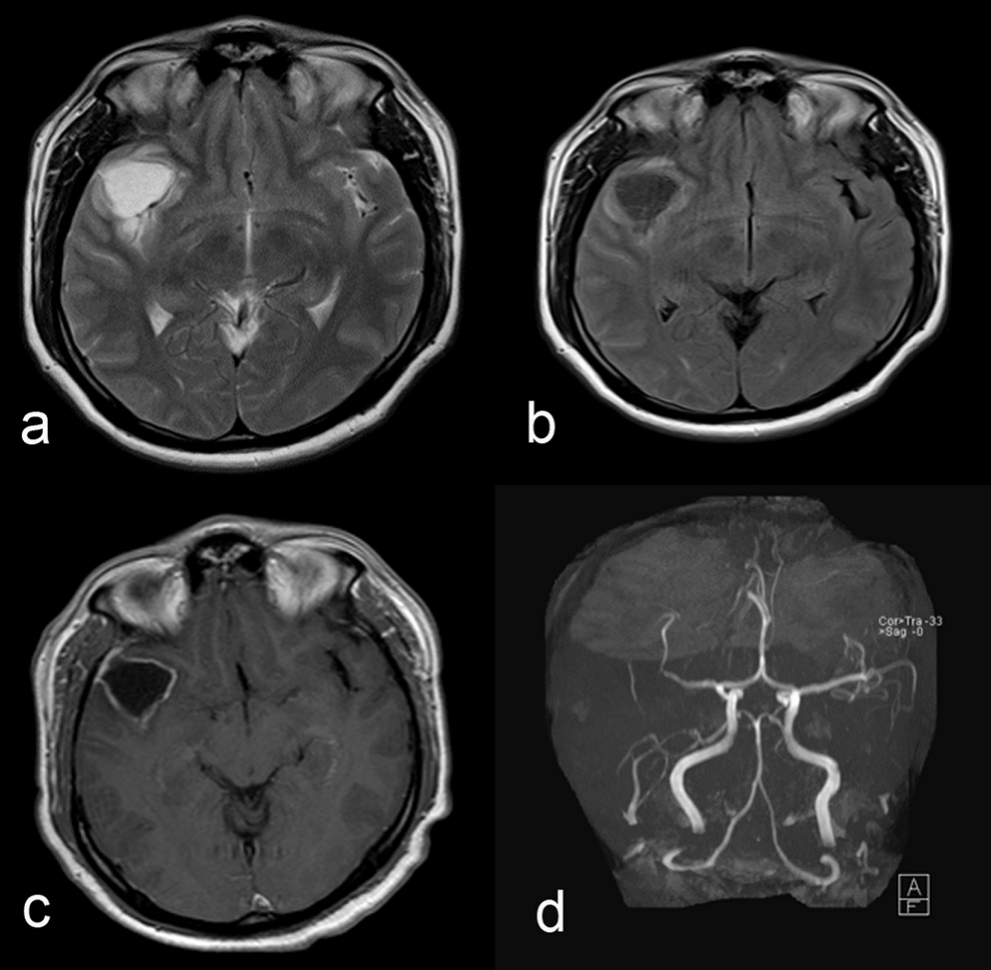
Axial *T*_2_ (**a**), fluid-attenuated inversion recovery sequence (**b**), axial post-contrast *T*_1_ (**c**) and maximum intensity projection MR angiogram (**d**) images of the brain. A *T*_2_ hyperintense cystic lesion is seen in the right sylvian cistern with marked mild perilesional oedema and peripheral rim enhancement following contrast administration. Severe stenosis of the right middle cerebral artery bifurcation and occlusion of the M2 segment is noted

**Figure 4.**
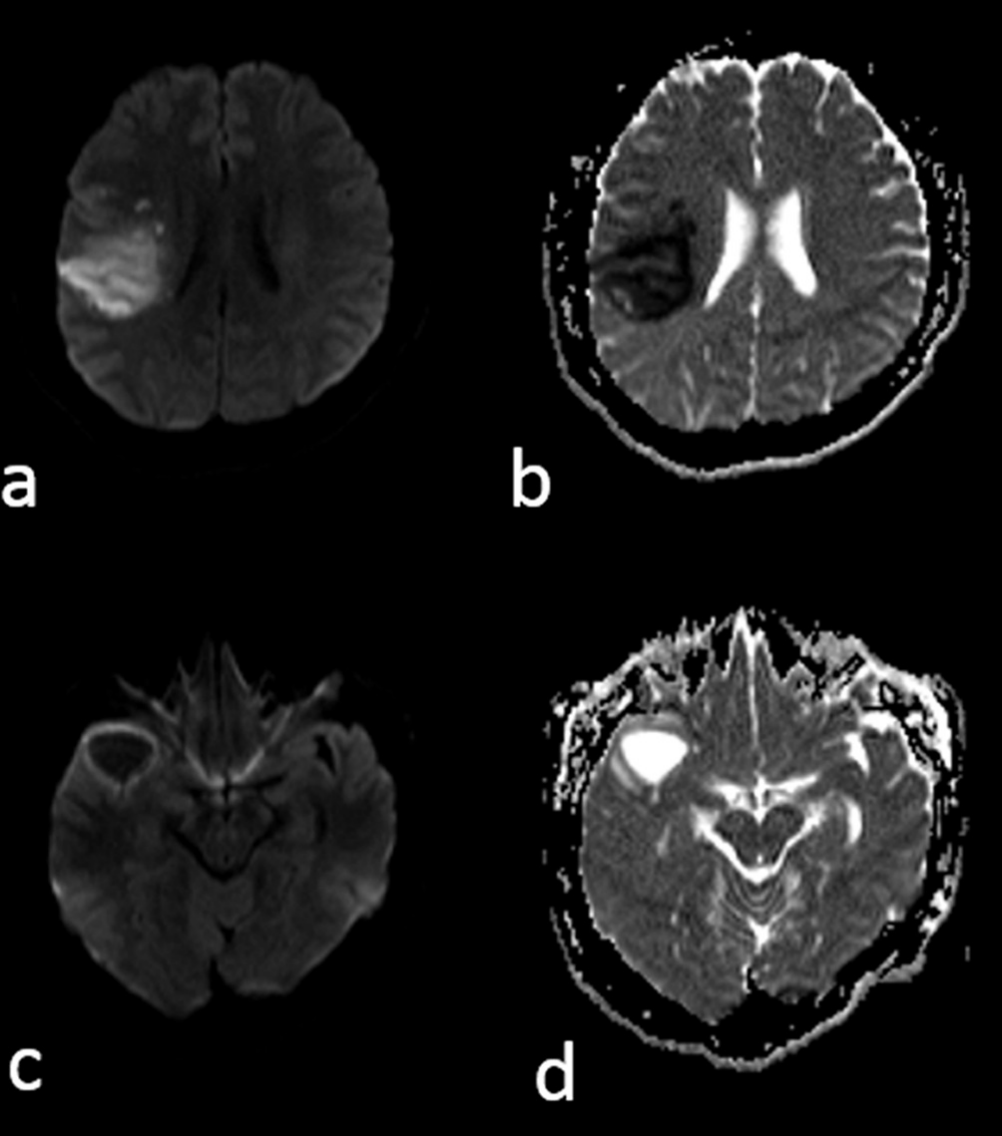
Diffusion weighted sequences (a, c) with corresponding apparent diffusion coefficient maps (b, d) show restricted diffusion in the right middle cerebral artery territory (a, b), consistent with an acute infarct. The cystic lesion does not restrict diffusion (c, d)

The patient underwent repeat head CT imaging, which demonstrated an acute right MCA territory infarct (Figure 2). The cystic structure seen previously remained unchanged. MRI and MR angiogram of the brain revealed luminal narrowing of the right MCA bifurcation and abrupt cut-off of one of the M2 branches (Figure 3d). The cystic lesion in the right sylvian cistern was adjacent to the infarcted territory and demonstrated signal characteristics not compatible with an arachnoid cyst, appearing hyperintense to the cerebrospinal fluid (CSF) on fluid-attenuated inversion recovery sequence (Figure 3b); demonstrating perilesional oedema and peripheral rim enhancement on post-gadolinium imaging (Figure 3c); and revealing no evidence of restricted diffusion (Figure 4c,d). Given its contiguity to the narrowed MCA, the lesion was suspected to represent an abscess or a neoplasm that caused extrinsic MCA stenosis and, ultimately, the MCA territory infarct.

## Treatment

The patient was admitted to the intensive care unit and started on aspirin, intravenous broad-spectrum antibiotics and seizure prophylaxis. Work-up for possible causes of stroke was unremarkable, which included electrocardiogram, echocardiogram, coagulation profile and autoimmune panel for vasculitides. Blood cultures showed no evidence of infection. Neurosurgical consultation was sought for biopsy of the lesion to guide treatment. In view of the patient’s demographics, the possibility of NCC was suggested, and serological markers for NCC were obtained. A lumbar puncture yielded unremarkable CSF results. On the second day after the onset of symptoms, the patient had worsening of his neurological deficit. Repeat head CT imaging demonstrated progression of cytotoxic oedema in the infarcted territory. Corticosteroid treatment was then initiated, to which the patient had a favourable response. Since neoplasm was a diagnostic consideration, the patient underwent stereotactic biopsy and cyst aspiration. His post-operative course was complicated by seizures, cerebral oedema and intracranial hypertension, requiring decompressive craniectomy and ventriculostomy catheter placement. Serological titres and western blot antibody test were consistent with cysticercosis and on the eighth day of hospitalization, the patient was started on antiparasitic therapy, comprising praziquantel and albendazole. Subsequently, biopsy results also confirmed NCC. The patient remained hospitalized for 5 weeks, received antiparasitic, intravenous steroids and anticonvulsant therapy.

## Outcome and follow-up

**Figure 5.**
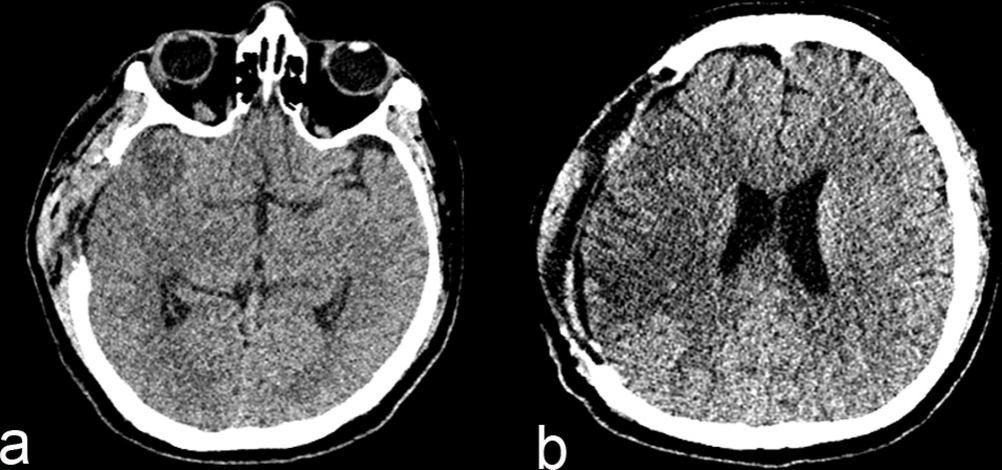
Non-enhanced CT scan of the head demonstrates post-surgical changes from right pterional craniectomy. Right middle cerebral artery territory infarct is noted with the cystic lesion previously noted in the right sylvian cistern decreased in size

Over the course of his hospital stay, the intracranial hypertension improved, the ventriculostomy catheter was removed, and steroids were tapered. Repeat head CT imaging prior to discharge showed a right-sided craniectomy defect and a large MCA territory infarct (Figure 5). The mass effect on the contralateral hemisphere had resolved. The patient completed a 4 week course of 400 mg albendazole and was discharged with mild left upper extremity weakness.

## Discussion

NCC remains the most common parasitic infection affecting the human nervous system.^1^ Over the last several years, there has been a steady increase in the incidence of NCC in the UK and the USA, owing to the rise in migration from endemic areas.^2,3^ During the past decade, NCC has been identified as an independent risk factor for stroke.^4^ Strokes attributable to the parasitic infestation may be seen in 2–12% of patients affected by NCC.^5,6^ In endemic areas, NCC is therefore not an uncommon cause of cerebrovascular disease in young and middle-aged populations, although its significance is often underestimated.^4^

The clinical spectrum of the disease is dictated by the cyst location, size, number, stage and the degree of the host’s immune response to the parasitic agent.^2,6,7^ Based on location, NCC is classified into four categories, of which subarachnoid is the most common, followed by the parenchymal, intraventricular and spinal forms.^6^ Subarachnoid NCC usually involves the basal cisterns.^8^ The subarachnoid form is most commonly associated with cerebrovascular complications,^2^ probably owing to the proximity of the cysticerci to vascular structures. A study found 53% of patients with subarachnoid NCC to have arteritis on cerebral angiography.^9^ Several mechanisms have been implicated in cerebrovascular events associated with NCC, including cysticerci-induced vasculitis, thrombosis of cortical vessels and occlusion of small cerebral perforators from chronic inflammation, formation of a mycotic aneurysm owing to weakening of the vessel walls and its subsequent rupture.^2,5,10^ Middle and posterior cerebral arteries are most often implicated, as in our patient.^9^

NCC may have a variety of radiological appearances, depending on the stage and viability of the parasite.^2,6^ The most commonly seen findings include cerebral calcifications that represent the nodular stage of the disease.^6,7^ In our patient, the cystic lesion in the right sylvian cistern had slightly higher attenuation than CSF on CT imaging and demonstrated marked peripheral enhancement on MRI. This appearance represents the vesicular stage of NCC, which can easily be confused with other infectious processes, especially in the absence of a scolex, which usually presents as a small, round isoattenuating structure within the cyst. It is worth noting that a scolex is typically absent when multiple cysts are present, a manifestation referred to as racemose NCC.^6,8^ In such circumstances, the patient’s demographics might increase the index of suspicion for NCC and, utilizing serological testing, one can establish an early and accurate diagnosis.^7^ Histological diagnosis is rarely necessary and can potentially lead to significant morbidity, as it did in our patient, prolonging his hospital stay.

Management of subarachnoid NCC is still unclear, with no controlled trials available to date on the treatment of this form of disease.^2,7^ Similarly, data on treatment of ischaemic cerebrovascular events in the setting of cerebral cysticercosis is scarce, limited to a few case series and reports.^2^ A few studies have proved the importance of antiparasitic drugs, steroids and shunting for hydrocephalus; however, the optimal dose and duration of antiparasitic therapy have not been established.^2^ Surgery, previously regarded as the primary form of treatment, is now reserved for patients who do not respond to medical management or develop life-threatening intracranial hypertension despite treatment with steroids.^1^ In patients with cerebrovascular complications, corticosteroids are considered the mainstay of treatment.^1,2^ Complete resolution of MCA stenosis has been reported with a 1-month course of steroids in a patient with NCC.^10^

In conclusion, NCC should be considered in the differential diagnosis for stroke in patients belonging to populations from endemic areas, especially in the young and middle-aged groups, who lack the typical risk factors for cerebrovascular disease. As migration from endemic areas to the UK and USA continues to increase, the frequency of NCC will increase further. Hence, it is essential that radiologists familiarize themselves with the myriad imaging manifestations of this disease entity in order to establish early diagnosis and treatment.

## Learning points

1. NCC is an independent risk factor for stroke and should be considered in the differential diagnosis in stroke patients belonging to endemic regions.
2. Radiologists should be aware of the variable imaging manifestations of NCC, including stages when the scolex is absent, as in the setting of racemose NCC.

## Consent

Informed consent was obtained from the patient to publish case history, images and data.

## References

[1] Proaño JV, Madrazo I, Avelar F, López-Félix B, Díaz G, Grijalva I Medical treatment for neurocysticercosis characterized by giant subarachnoid cysts. N Engl J Med 2001; 345: 879–85. 11565520 10.1056/NEJMoa010212

[2] Garcia HH, Del Brutto OH, Nash TE, White AC, Tsang VC, Gilman RH New concepts in the diagnosis and management of neurocysticercosis (*Taenia solium*). Am J Trop Med Hyg 2005; 72: 3–9. 15728858

[3] Hawk MW, Shahlaie K, Kim KD, Theis JH Neurocysticercosis: a review. Surg Neurol 2005; 63: 123–32. 15680651 10.1016/j.surneu.2004.02.033

[4] Alarcón F, Vanormelingen K, Moncayo J, Viñán I Cerebral cysticercosis as a risk factor for stroke in young and middle-aged people. Stroke 1992; 23: 1563–5. 1440703 10.1161/01.str.23.11.1563

[5] Alarcón F, Hidalgo F, Moncayo J, Viñán I, Dueñas G Cerebral cysticercosis and stroke. Stroke 1992; 23: 224–8. 1561652 10.1161/01.str.23.2.224

[6] Kimura-Hayama ET, Higuera JA, Corona-Cedillo R, Chávez-Macías L, Perochena A, Quiroz-Rojas LY, *et al* Neurocysticercosis: radiologic-pathologic correlation. Radiographics 2010; 30: 1705–19. 21071384 10.1148/rg.306105522

[7] Cantey PT, Coyle CM, Sorvillo FJ, Wilkins PP, Starr MC, Nash TE Neglected parasitic infections in the United States: cysticercosis. Am J Trop Med Hyg 2014; 90: 805–9. 24808248 10.4269/ajtmh.13-0724 PMC4015568

[8] Hauptman JS, Hinrichs C, Mele C, Lee HJ Radiologic manifestations of intraventricular and subarachnoid racemose neurocysticercosis. Emerg Radiol 2005; 11: 153–7. 16028320 10.1007/s10140-004-0383-y

[9] Barinagarrementeria F, Cantú C Frequency of cerebral arteritis in subarachnoid cysticercosis: an angiographic study. Stroke 1998; 29: 123–5. 9445339 10.1161/01.str.29.1.123

[10] Bouldin A, Pinter JD Resolution of arterial stenosis in a patient with periarterial neurocysticercosis treated with oral prednisone. J Child Neurol 2006; 21: 1064–7. 17156699 10.1177/7010.2006.00132

